# Long-term evaluation of patient-specific carbon fiber implants for calvarial defects

**DOI:** 10.1007/s00508-025-02614-7

**Published:** 2025-09-19

**Authors:** Michael Rasse, Phillipe Dodier, Christoph Sacher

**Affiliations:** 1 Medical University of Innsbruck, Christoph Probst-Platz 1, 6020 Innsbruck, Austria; 2 Department of Neurosurgery, Medical University of Vienna, Währinger Gürtel 18-20, 1090 Vienna, Austria; 3 Department of Oral and Maxillofacial Surgery, Medical University of Vienna, Währinger Gürtel 18-20, 1090 Vienna, Austria; 4 Tegetthoffstraße 15, 4020 Linz, Austria

**Keywords:** Calvarial-implant, Implant-prefabrication, Imbedded carbon-fibres, Implant-evaluation, Long-term outcome

## Abstract

**Background:**

Preformed implants based on computed tomography (CT) data have widened the spectrum of calvaria reconstruction. Different materials have been used. Carbon fiber-reinforced resin implants were first introduced into calvaria reconstruction in 1996.

**Method:**

A long-term study of such implants including subjective comfort data as well as a radiological control of adjacent tissues and a thorough clinical investigation seemed of value to see whether or not the implants were integrated and whether there were long-term complications.

From a collective of 27 patients with calvarial defects surgically treated at least 20 years ago with insertion of such an implant, 9 patients could be followed up. A neurological and maxillofacial status was performed, The CT scans were obtained to assess the implant fit, changes in surrounding tissues and the brain. A health-related quality of life evaluation was performed.

**Results:**

There was no implant loss or operative revision. No infection was experienced. Patient satisfaction was maximum in all cases. The stated restrictions in quality of life in the case histories, impaired esthetics, pain and dysesthesia, were completely cured after implantation and were permanent over more than 20 years. There were no neurological pathologies evoked by the reconstruction, except a slight dysesthesia along the coronal scar and a minor muscular atrophy of the temporal muscle in one case each. No other functional deficits were detected. The implant fit was undisturbed without any implant loosening or resorption of bone.

**Conclusion:**

Carbon fiber-reinforced resin implants seem a valuable option for calvarial reconstruction. The material proved to be biologically inert.

## Introduction

Reconstruction of cranial vault defects has been performed with various techniques. Autologous material was variably used: bone dust shed on the dura mater [36], split diploë [20], ribs [23], cartilage [33], Autolysed antigen-extracted allogeneic(AAA)-bone or replantation of refrigerated bone [17, 28] and vascularized bone flaps [24]. These defects especially in less plane areas and on the forehead may cause problems for autologous implants. Complications become more likely when the size of the graft increases. Sinus bleeding, venous hemorrhage and also different amounts of resorption of replanted bone have been reported [13, 28].

To overcome the drawbacks of autologous implants, primarily their availability and possible resorption, alloplastics have been introduced, reviewed and compared.

Polymethylmethacrylate [41] has been used as a reconstruction material for decades. Polymethylmethacrylate, widely used to intraoperatively form a coverage for calvarial defects, has several drawbacks: heat production while polymerizing, toxicity of monomer and catalyst, moisture uptake and hydrolysis [2] and aging. Furthermore, complex forms of transplant are hard to shape intraoperatively. Other alloplastics that have been reported are titanium mesh, printed titanium [14, 41], printed titanium with ceramics [26], polyetheretherketone (PEEK) [14, 32].

Secondary reconstruction is the prevailing procedure, although primary reconstruction, e.g., in cases of possible planning of the exact size and shape of resection as in fibrous dysplasia, has been reported [14, 15] and has also been performed by the first author of this article.

In 1996 prefabricated carbon fiber implants for use in cranioplasty were first brought to the public knowledge [31] and also published in 2002 [35].

Many types of polymer matrices with carbon fiber reinforcement have been developed since and 20 of them have been tested for the acute and chronic toxicity, mutagenicity and biocompatibility with favorable outcome [11]. Carbon fibers embedded in EPN/DDS (epoxy resin and dicyandimide as hardener, EPN, CAPROMAN® by MAN Company, Deggendorfer Werft- und Eisenbau GmbH, Deggendorf, Germany; succesor Medtronic Sofamor Danek Deggendorf GmbH, Deggendorf, Germany) have been used in orthopedics for acetabular cups and hip stems [8]. Other carbon fiber-reinforced materials have been used for spinal application (lumbar interbody fusion cages, composite cages for cervical spine, rods, anterior cervical plating systems [16]).

Retrospective studies on alloplastic material for implants have so far only covered a limited period of time. A long-term 20 years retrospective overview of calvarial implants therefore seems to be of interest.

## Material and method

Carbon fiber-reinforced plastics (CFRP) have a great strength associated with lightness (their specific weight is 20% that of steel). The modulus of elasticity is close to that of bone [4, 6, 7, 9, 10]. They are radiolucent. By selecting the type of the fibers (length and thickness) and their orientation, mechanical properties can be varied [18, 37]. Fibers have a much higher elastic modulus longitudinally and a much higher strength then the matrix but they can only display these advantages as a compound material where the fibers are protected and shearing forces are constrained. Biocompatibility in general and tolerance in bone tissue in particular, has been proven for the fibers and the epoxy-matrix [1, 10, 12, 19, 29, 39].

High durability is guaranteed and well tested with mechanical testing facilities and in animal experiment (5.5 years of animal testing time for hip joint replacement [5]), tests for genotoxicity (DNA repair test, reverse mutation assay) and cytotoxicity (direct contact procedure, agar diffusion test, extraction procedure, scanning electron microscopy SEM, growth inhibition test), short-term and long-term implantation assay (unpublished data, MAN Company, Deggendorf, Germany).

The material causes no artefacts in CT or magnetic resonance imaging (MRI) and bone changes in the contact areas can easily be detected. The material causes no scattering in cases of tumor irradiation.

The implant can be sterilized several times. If necessary, the model can be ground in the operating theater.

The material can be used for prefabricating an implant, if the desired form is exactly known. With the stereolithographic skull models this requirement is fulfilled. To exactly shape the model that replaces a missing part of the skeleton a mirror image model can be built by transferring the data from one side to the other (Fig. 2). Therefore, the intact contralateral side can be used to build a fitting part. The stereolithographic “transplant” is then built in carbon fiber-reinforced epoxy resin. This model not only recontours the outer surface anatomically but also displays an anatomically correct inner surface. This seems to be of special importance if parts of the skull base are involved. The positive templates are used to build moulds and fabricate the implants at Medtronic-Sofamor Danek Comp. (Medtronic-Sofamor Danek, Deggendorf, Germany). The CFRP medical grade implants are manufactured by embedding carbon fibers into a thermoset epoxy resin matrix (EPN/DDS). Depending on required mechanical properties, two types of fibers are used. The first part is a woven fabric of polyacrylnitrile (Ø 5.2 µm). These carbon long-chain fibres with high tensile mechanical properties improve the strength especially at the bone-implant interface where fixation screws and plates are used. The place of the holes for the fixation screws is designed by the surgeon and burred at the end of the production as are the perforations for the dura fixation sutures. The second, chopped fibre material (length 200 µm, Ø 13 µm) is used to mould the implant into the required shape. The implants are produced under ISO9001. No softening agents, catalysts, or stabilizers are used. The polymerization of the resin lasts for 16 h at 170 °C. Finally, the remaining humidity and residual monomers are removed by an degassing treatment in high vacuum (120 °C, 72 h). The material is biocompatible, tested according to ISO10993 (data from Medtronic-Sofamore® and [35]).

### Clinical applications

**Fig. 1:**
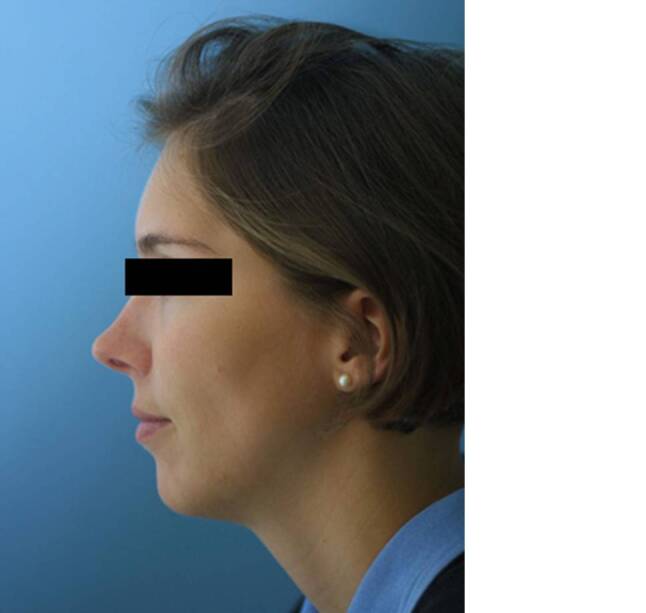
Fibrous dysplasia of the frontal bone, resected and immediate reconstruction with carbon fiber implant. Patient after surgery

**Fig. 2:**
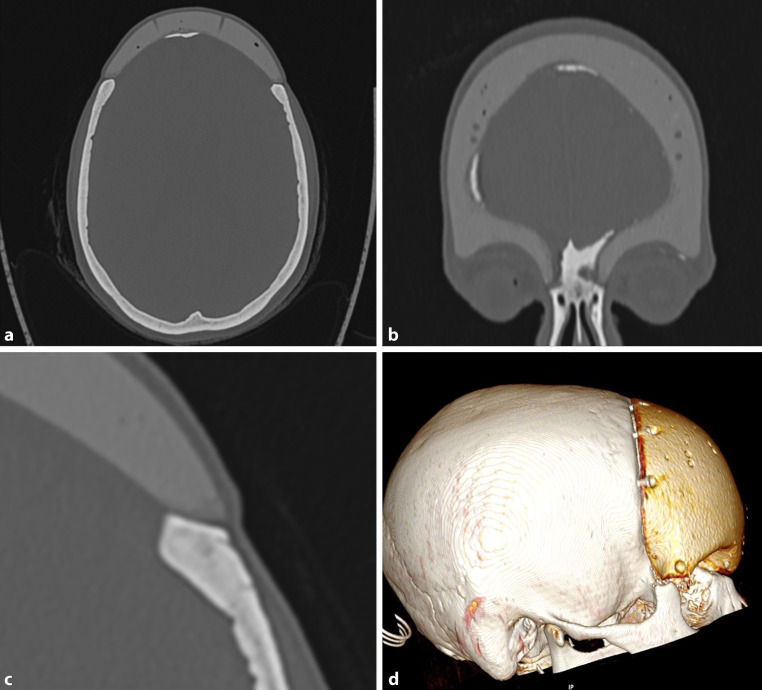
CT scans of a carbon fiber-reinforced resin implant after resection of a fibrous dysplasia of the frontal bone, 22 years after implantation. **a** This scans show an Axial view, the calcification of adjacent dura, bore holes in the implant for suturing the dura to the implant. **b** Same patient in a Coronal view. **c** The axial view shows sufficient implant-bone contact. **d** 3‑D reconstructions shows the positioning of the screws

**Fig. 3:**
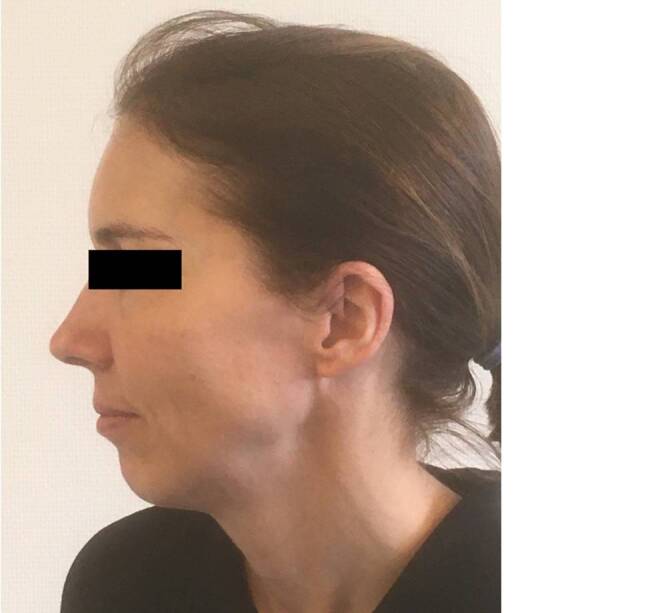
Same patients as in Fig. 1; 22 years after carbon fibre implantation, no obvious changes

**Fig. 4:**
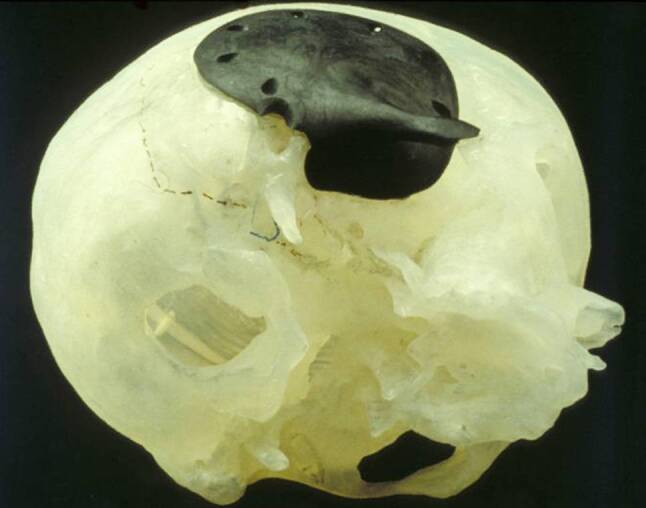
Fibrous dysplasia of the temporal bone, prefabricated carbon fiber implant on a 3D model with preformed bore holes for lag screws

**Fig. 5:**
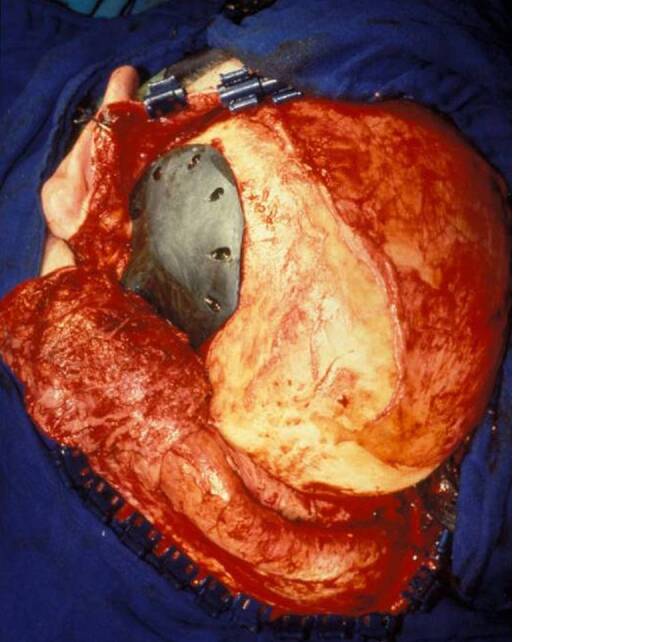
Intraoperative situs

**Fig. 6:**
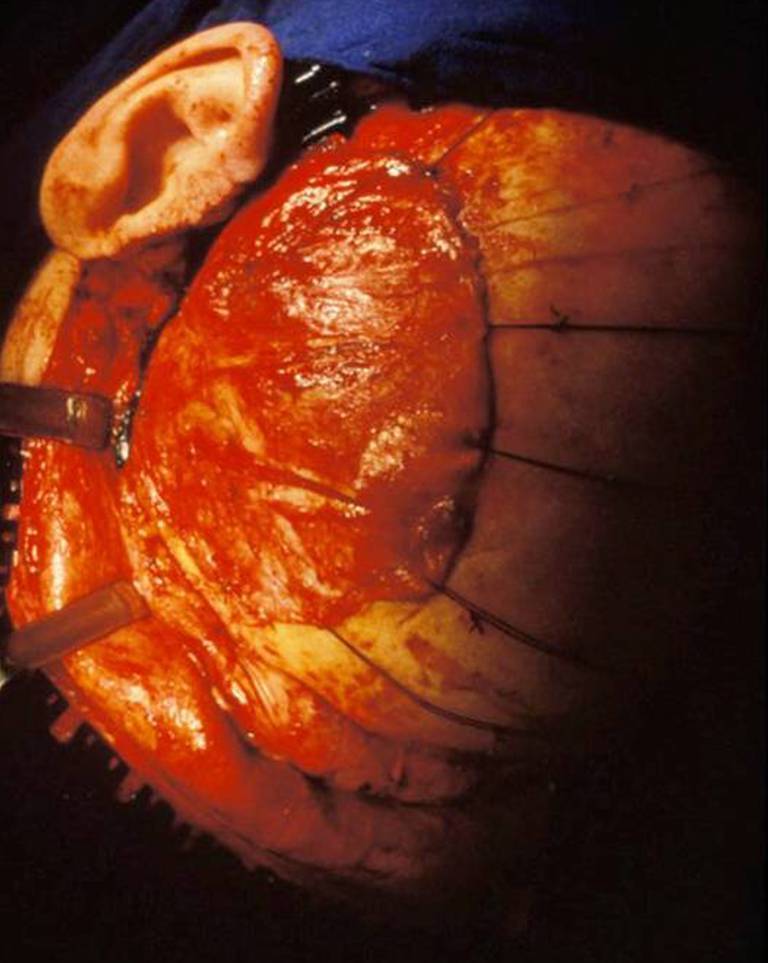
Covering the implant with the temporal muscle

This technique was used for secondary reconstruction of calvarial defects in 25 cases and in 2 cases for primary reconstruction (Figs. 1, 2, 3, 4, 5 and 6). The patients underwent reconstruction after surgery for trauma, bleeding, osteomyelitis and tumors.

Access to the site of defects was gained by employing the incision used in primary surgery, mostly a coronal incision. The periosteum was incised 1cm laterally to the defect, sheared off the bone at the rim of the defect and left in continuity with the dura mater. As the stereolithographic model used for building the implant is a model of the skull bone, the soft tissue has to be stripped off completely. If the temporal muscle was lifted, it was refixed to the periosteum of the undisturbed skull and to bore holes of the implant at the end of surgery (Fig. 6).

Dura reconstruction with periosteal flaps had to be performed two times. In two cases it was necessary to reduce intracranial pressure by application of mannitol in order to properly place the implant. In the cases of the primary reconstruction small details of the implant had to be recontoured with diamond fraises. Titanium screws applied through prefabricated holes were used to fix the implant. In places where the adjacent bone could not host a lag screw, miniplates were used.

If the form and amount of bone resection can be predicted, primary reconstruction is possible.

Meticulous care was taken to cover any paranasal sinus or the mastoid cells with vascularized soft tissue flaps to avoid open contact of the implant to an “outer surface”. In five cases the frontal sinus or its duct was filled with bone and isolated from the implant. An i.v. antibiotic prophylaxis administered, starting with a preoperative dose of amoxycillin 1000 mg/clavulanic acid 100 mg which was applied 3 times a day for 3 days.

### Long-term control

The primary objective of the study was to evaluate the quality of life and subjective judgement of the patient after carbon fiber calvarial implants more than 20 years after implantation.

The change of quality of life in relation to the preoperative status was checked with a questionnaire. The satisfaction with the treatment and restrictions by the implant and disturbances in daily life were checked.

The secondary objective was the evaluation of neurologic sequelae and radiologic changes of tissues surrounding the implant. The change in quality of life in relation to the preoperative status was checked with two questionnaires. The first asked for satisfaction with the treatment. It was assessed on a scale (1–10). This comprised the treatment satisfaction on the whole, fulfilment of the patients’ expectations, restriction of quality of life prior to reconstruction and postoperative restrictions. The second questionnaire asked for specific subjective data, answered by the patient with yes or no, including exposure of the implant, experienced pain, foreign body feeling, disturbance of sensitivity or sense of temperature and general restrictions in daily life.

A general neurologic status was established, and a specific maxillofacial status was assessed including a test of the cranial nerves.

To control the fit of the implants a CT was employed (Siemens Somatom Sensation 64 CT Scanner®. Scans were acquired at 120 kV, mean X‑ray tube current 288 mA with a field of view of 23 cm and slice thickness 0.6 mm) and three planes were used for the linear measurements. For calculating the surface area the CT data were transformed into surface files. The software 3D Slicer® was used. The distance of the implant to the adjacent bone was measured at the maximum distance, further the maximum distance of the implant to the dura and the width of the subdural space perpendicular to the implant. It was controlled whether the screws stood out of the implant on the inner surface and possibly perforated the dura. The structure of the brain under the implant was compared to the preoperative status and whether there were changes.

## Results

Of the 27 operated cases 9 could be controlled after more than 20 years and were prospectively included in the study. We present a mean follow-up of 23.3 years.

The age at reconstructive surgery showed a mean of 36.5 years, a minimum of 20 years and a maximum of 52 years. The implants had been in place for 23.3 years (mean), with a minimum of 20 years and a maximum of 26 years.

No implant of the controlled patients had to be removed or replaced. There was no report in the patient data of a necessary implant removal of those patients that were not controlled in this study and had been operated on by the first author.

The reasons for the surgical resection of the calvaria and the following defects were bleeding of an arteriovenous malformation 2×, trauma 2×, tumor 2×, fibrous dysplasia 1×, aneurysmal bleeding 1×, sinusitis frontalis osteomyelitis 1×.

No patient was a smoker or had been a smoker. There was no patient with diabetes mellitus. No patient had been administered cortisone in the time after reconstruction. No patient had had osteoporosis or any kind of treatment concerning such pathologies.

### Evaluation of subjective data and CT results

The results of subjective data are given in Table 1 and 2, the results of the CT scans are shown in Table 3.

**Table 1:** Average satisfaction with treatment results (rating scale 1–10)

	Scale 1–10
Treatment satisfaction	Mean 10
Expectations fulfilled by surgery	Mean 10
Restrictions in quality of life prior to reconstruction	Mean 8.38, min 6, max 10
Postoperative restrictions in quality of life	0

The preoperative restrictions in the quality of life were caused by unfavorable esthetics 7×, pain 3×, dysesthesia 4×. Postoperative limitations in quality of life were given in addition to the neurological deficits already present due to the underlying disease.

**Table 2:** Average satisfaction with treatment results (rating scale 1–10)

	Yes	No
Restrictions by the implant in daily life	0	9
Visible exposure of the implant	0	9
Pain in the area of the implant	0	9
Recurrent swelling in the area of surgery	0	9
Foreign body feeling	0	9
Subjective disturbance of sensitivity in the operated area	1	8
Sensitivity to temperature	0	9

The patient with reduced sensitivity in the right half of the calvarai had undergone tumour resection, which was the cause of this disorder.

**Table 3:** CT findings

	Yes	No
Implant loosening	0	9
Dura perforation by screws	0	9
Calcification of dura under the implant	6	3
Epidural space changed after reconstruction	0	9
Subdural space changed after reconstruction	0	9
Parenchymal changes after reconstruction	0	9
Primary brain lesions	6	3

### Implant-specific data

Size of implant: mean 118.3, minimum 14.85, maximum 451 cm^2^.

Maximum distance between implant and bone in mm: mean 1.88, minimum 0, maximum 5.

Number of screws for fixation: mean 9.3, minimum 3, maximum 22.

Number of plates for fixation: in 4 patients 1–7 plates were used.

There was no intracranial prominence of screws.

The minimum distance of the dura from the implant in mm was mean 1.96, minimum 0, maximum 4.

### Maxillofacial and cranial nerves status

N I: no patient showed anosmia.

N II: 1 patient showed pupil disturbance (width and reaction) and restrictions in the visual field after the primary disease, prior to reconstructive surgery.

No patient showed worsening of visual acuity and worsening of the results of a finger perimetry, pupil reaction, double vision, width of palpebral fissure, ocular movement or dislocation of the ocular bulb after reconstruction.

N III, IV, VI were without pathologic findings.

N V: 1 patient had an impairment of sensitivity in all branches of the trigeminal nerve after the primary bleeding. The primary loss of sensitivity improved over the first 1.5 years. There was no motor weakness of this nerve detectable.

After reconstruction one patient had a slight dysesthesia in the area of the coronal incision. All other parameters were normal.

N VII: 1 patient had a primary motor deficit. No additional deficit was observed in a patient after reconstruction.

N VIII–XI: were without pathologic findings.

N XII: 1 patient showed a mild motor deficit after the primary event. No other pathologies were detected.

The greater and lesser occipital nerves were without pathologic findings.

#### Atrophy of muscles

Temporal muscle: 1 patient showed some atrophy over the implant, the frontotemporal soft tissues were slightly reduced.

The frontal and temporoparietal muscles showed no pathology in movement or bulk.

One patient showed a very moderate loss of hair in the implant region.

There were no noticeable asymmetries of the head or other abnormalities.

In two cases the rim of the implant could be palpated.

### Neurologic status

A neurologic status was assessed and included state of awareness, orientation, pain, meningism, speech, visual field, pupils, nystagmus, sensitivity, velar movement, arm holding attempt, finger-nose test, fine motor skills, diadochokinesis, strength elbow, grip, finger flexor reflex, snap reflex, biceps reflex, radioperiosteal reflex, triceps reflex, sensitivity C5-Th1, abdominal reflex, motor leg, muscular strength of lower extremity, toe movement, knee jerk reflex, Achilles tendon reflex, pyramidal sign, sensitivity lower extremity, Romberg test, Unterberger test, palmomental reflex, snout reflex, tiptoe standing, gait, gait ataxia, stand ataxia, fasciculation, cranial nerves.

No patient had any pathology concerning state of awareness and orientation. No patient showed meningism.

There was no sign of any neurologic pathology that had evolved after the reconstructive surgery. There were sequelae of the primary disease, which will not be addressed here.

## Discussion

The permanent fit of the implant depends on the biocompatibility and integration of the implant. Biocompatibility in general and tolerance in bone tissue in particular has been proven for the fibers and the epoxy matrix as well [19]. It was reported that carbon fiber-reinforced composite stimulates osseointegration which was also attributed to its semi-antioxidant properties [30]. High durability is guaranteed and well tested with mechanical testing facilities and in animal experiment (5.5 years of animal testing time for hip joint replacement) [5]. By selecting the type of the fibers (length and thickness) and their orientation, mechanical properties can be varied [18, 37]. The biocompatible properties were also confirmed clinically in traumatology [38] and orthopedics for acetabular cups and hip stems [8]. It was shown that it even enhances wound healing in soft and hard tissue injuries [3]. These reported advantages probably form the basis for the results of this study in addition to the chosen surgical procedure.

Many review articles dealt with the comparison of the outcome of cranioplasty using autogenous materials, mostly bone and alloplastic (e.g., bone versus biomaterials [27], titanium mesh versus autologous material [11], 3D-printed calcium phosphate implants versus traditional materials [22]). Total failure, bone resorption, infection, wound dehiscence, cerebrospinal fluid leakage and esthetic problems are the major concerns in these papers. The reported infection rate varies from 0–30% [40]. The reported total failures for 60 patients, e.g., with either 3D-printed calcium phosphate, alloplastic or autologous transplants were 14 (19.7%) within an average control time of 4 years, with no significant differences between the goups [22]. A recall at 2–5 years [21] of 38 patients with 40 PEEK implants showed a 28% complication rate, including 13% infections and 2.5% with a cerebrospinal fluid (CSF) leak. The infected implants were removed, sterilized and implanted again. A 3‑center study of 66 cranioplasties over 2–10 years [32] reported a 7.6% infection rate and 9.1% removal rate for PEEK implants. There are other reports without infection or removal of implants with ceramic e.g., [25], a 5-year control of 4 cases of PEEK implants, 6 cases of Palacos reaction and 14 cases of replanted bone showed no necessity for revision [34]. The same complication-free results were reported for PEEK [15].

In our study there was no implant loss and none of the treated patients who did not come to the recall had been operated on a second time in the clinics involved. A major cause of complications is infections. The isolation of the implant from any direct contact to the nasal cavity, paranasal sinuses or mastoid cells that has been observed in our cases, seems to be of importance. No infection was seen at any time of the postoperative course. This is in contrast to many of the cited reports [21, 32, 40].

The quality of life of the patients significantly improved from the prereconstruction status to the status after carbon fiber implant insertion. This is clearly shown by the fact that no patient suffered any restriction in the quality of life after the reconstruction (0 of 10 possible points in all cases), whereas prior to reconstruction only 3 patients had no restriction and the others varied from 6–9 points in their appraisal. This also confirms the positive effect of reconstruction to overcome the syndrome of the trephined (SoT) as also reported in a previous study [20]. All patients were completely satisfied with the result and their expectations were fulfilled. The objective parameters confirmed the personal assessment.

There were no restrictions reported in daily life, no foreign body feeling. There was no pain in the reconstructed area or loss of sensitivity. This could be a sequela of the surgery or a reaction to the implant. Only one patient complained of occasional dysesthesia in the area of the coronal incision. The muscles innervated by the facial nerve (temporoparietal and frontalis muscles) showed normal movement. This shows that there is no impeding scar formation over the implant. A gliding zone is re-established.

**Fig. 7:**
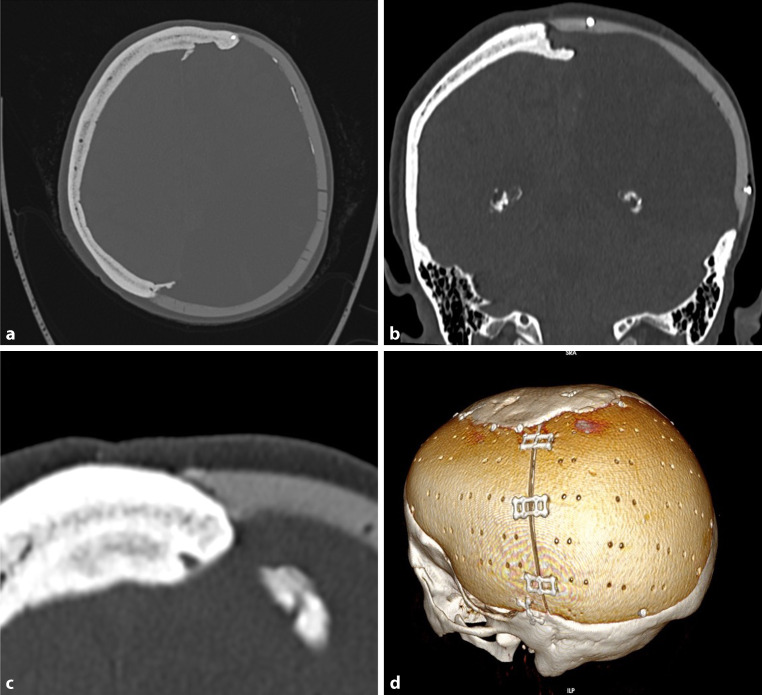
CT scans of reconstructed calvaria after meningeoma resection, 21 years after reconstruction; **a** Axial view, calcification of dura; **b** Coronal view, bone formation under the implant; **c** Bone-implant contact; **d** 3‑D image of the 2 implant pieces

The fit of the implant was satisfactory (Fig. 2 and 7) and preserved over more than 20 years as the measurements of the maximum bone-implant distance show (mean 1.88 mm). There were no signs of implant loosening clinically or in the CT. All osteosynthesis material was tightly fixed to the implant and bone. There was no screw that showed a prominence into the intracranial space and no dura involvement. Therefore, the primary measurements for the screws were correct and there was no bone resorption round the osteosynthesis material or adjacent to the implant. This is also due to the possible preoperative planning of the screw length.

There were no parenchymal changes of the brain due to the reconstructive surgery or the implant and no pathologies of the meninges caused by the implant in the 20 years control.

The dura showed calcification under the implant in 6 of 9 cases (Fig. 2).

**Fig. 8:**
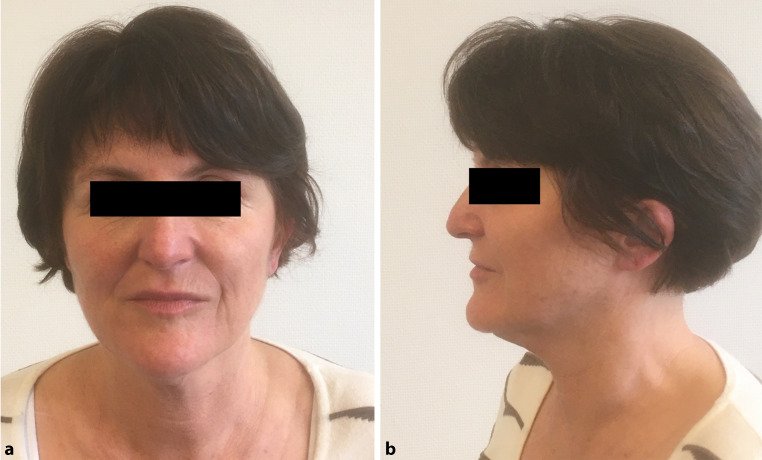
**a** Frontal picture of a patient with resection of a meningeoma and reconstruction with a carbon fiber implant of 451 cm^2^ in 2 pieces. 21 years after surgery; **b** Profil picture of the same patient.

The size of the implants varied from 14.85 cm^2^ to 451 cm^2^ (Figs. 8 and 7), with a mean of 116.3 cm^2^. The implant with the maximum size was inserted in 2 parts and crossed the midline (Fig. 7). Generally, the proper form could be achieved through the preoperative planning including mirroring technique. In none of the cases was it necessary to cancel the surgery because of an incorrect fit of the implant. As these reconstructions were done in major defects, the proper form would be very difficult to achieve with any material formed during surgery.

In cases of necessary relocation of the temporal muscle a concern was whether the reinsertion in its field of origin would be sufficient or if it would atrophy to a visible amount. In one patient a slight hollowing dorsal to the orbit could be detected. This might also be a sequela of incorrect positioning of the muscle in the primary surgery. The muscle was laid on the dura without stretching it sufficiently and shortened this way. As this was observed in one case only, it can be concluded that the muscle sufficiently inserts on the fascia or gains insertion on the implant as well. Chewing was unimpeded as well as jaw opening. Both criteria also show muscle preserving surgery at the first time.

One patient showed a minimally increased hair loss on the implant side compared to the other side. In all other cases the implantation did not cause hair loss as assessed by the patient or the controlling person.

## Conclusion

So far, no immediate or late complications have been experienced with carbon fiber implants in a recall of patients surgically treated at least 20 years ago. There was no sign of infection. No implant had to be removed. The implants have been mechanically stable. In cases of relocating of the temporal muscle, normal function could be proven clinically.

Loss of muscle substance due to atrophy must be taken into account dorsally to the lateral orbital wall.

The cosmetic results were satisfactory.

Even large bilateral defects could be correctly recontoured.
